# Ectopic Breast Cancer in the Axilla After Bilateral Breast Cancer Surgery: A Case Report and Literature Review

**DOI:** 10.7759/cureus.78639

**Published:** 2025-02-06

**Authors:** Hiroyuki Ishige, Yimi Mukouyama, Taijiro Kosaka, Satoshi Shiozawa, Noritoshi Sato

**Affiliations:** 1 Department of Breast Surgery, Saku Central Hospital, Saku, JPN; 2 Department of Pathology, Saku Central Hospital, Saku, JPN

**Keywords:** ectopic breast cancer, ectopic breast tissue, pectoral breast cancer, third primary breast cancer, us findings

## Abstract

Ectopic breast cancer (EBC) is a rare condition, and there have been no reports of axillary EBC as a third primary breast cancer after bilateral pectoral breast cancer (PBC) surgery. A woman presented with a mass in her axilla, which was located just beneath the skin. On ultrasound (US) examination, it appeared oval-shaped with uniform internal echoes and relatively clear boundaries. Pathologically, it was an oval-shaped, well-defined tumor and was diagnosed as encapsulated papillary carcinoma with invasion. EBC rises directly under the axillary skin, where axillary ectopic breast tissue exists. This area is not generally included in the resection field during mastectomy and axillary lymph node dissection, as illustrated in this case. US findings of EBC do not consistently show an irregularly shaped mass with indistinct boundaries, as these depend on the tumor's histological structure, similar to PBC.

When axillary masses are observed, it is crucial to consider the possibility of EBC based on their superficial location in the axilla and to predict histological architecture and type from US findings, similar to the approach for PBC.

## Introduction

Ectopic breast cancer (EBC) is a rare condition, accounting for 0.3% to 0.6% of all breast cancers [1], and the occurrence of both pectoral breast cancer (PBC) and EBC, representing a multicentric cancer, is extremely rare [2-5]. There have been no reports of axillary EBC as a third primary breast cancer after bilateral PBC surgery. Owing to its low frequency, axillary EBC is poorly recognized, leading to delayed detection in many cases [6,7]. Additionally, for patients who have undergone breast cancer surgery, a differential diagnosis of breast cancer recurrence is also necessary [8]. Here, we report the clinicopathological features of axillary EBC in a case with a history of bilateral breast cancer surgery, with a particular focus on the correlation between ultrasound (US) findings and histological architecture.

This article was previously posted to the Research Square preprint server on April 10, 2024.

## Case presentation

A 69-year-old Japanese woman with a history of bilateral breast cancer had a lump in her left axilla during postoperative follow-up. This patient was diagnosed with left breast cancer at the age of 41, and she underwent mastectomy and axillary lymph node dissection. Pathological examination revealed invasive ductal carcinoma measuring 1.8 cm, which was estrogen receptor (ER)-positive, progesterone receptor (PR)-positive, human epidermal growth factor receptor type 2 (HER2)-unknown and negative for lymph node involvement. She received postoperative hormone therapy. At 52 years of age, she underwent breast-conserving surgery along with sentinel lymph node biopsy for the diagnosis of right breast cancer. Pathological examination revealed an invasive ductal carcinoma measuring 0.7 cm, which was ER-positive, PR-positive, HER2-negative, with negative margins and no lymph node involvement. She received radiotherapy and hormone therapy postoperatively. The patient had no prior history of ovarian cancer and no family history of breast cancer or other malignancies.

**Figure 1:**
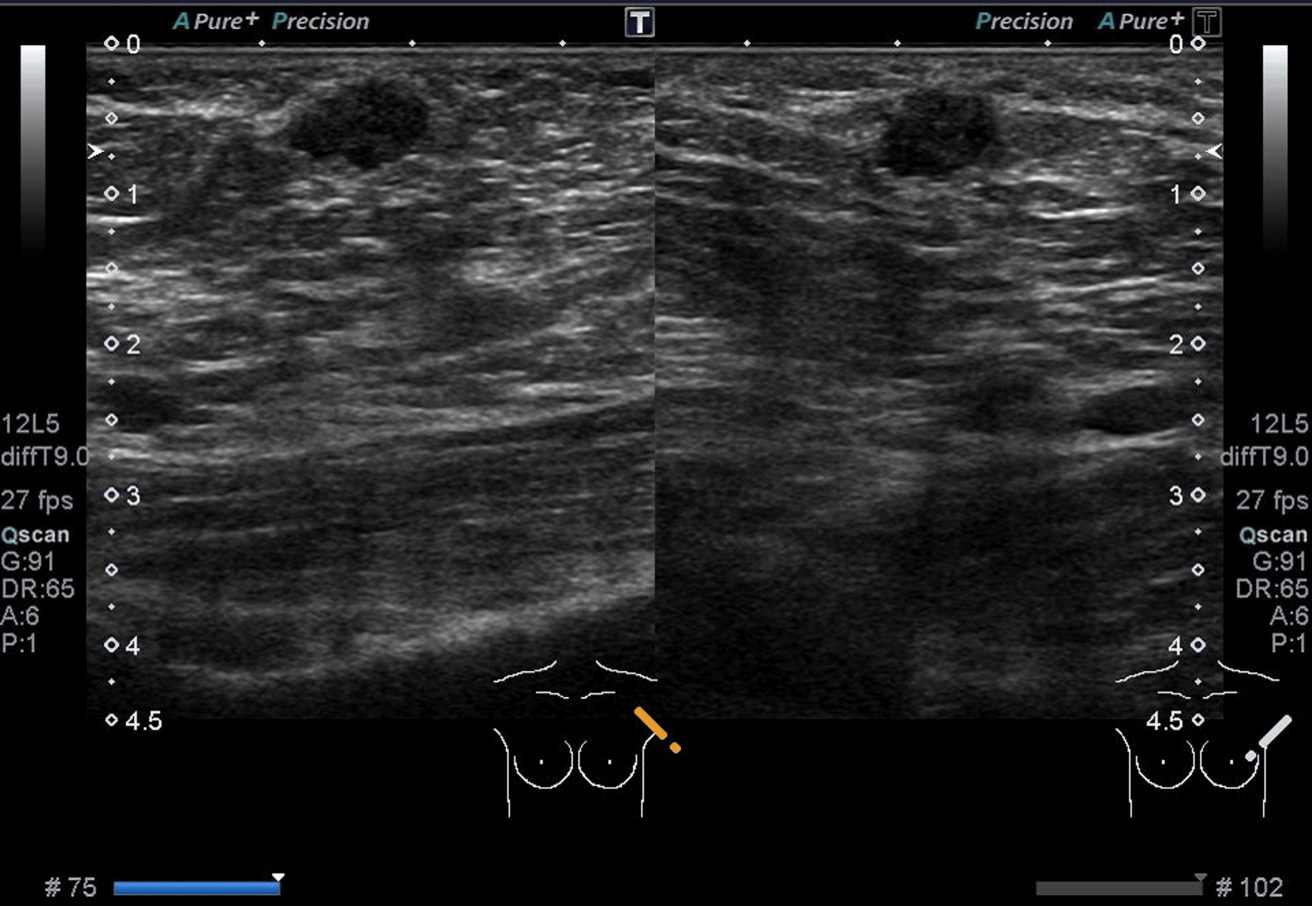
Sonography showing an oval, homogenous, relatively well-defined, hypoechoic mass in the left axilla, measuring 0.9 cm.

In her left axilla, a mobile but hard mass 1.0 cm in size was palpated. US revealed an oval-shaped hypoechoic mass located just beneath the skin, measured 0.9 × 0.9 × 0.6 cm, with relatively homogeneous internal echoes and well-defined borders (Figure 1). The patient was scheduled for regular check-ups.

**Figure 2:**
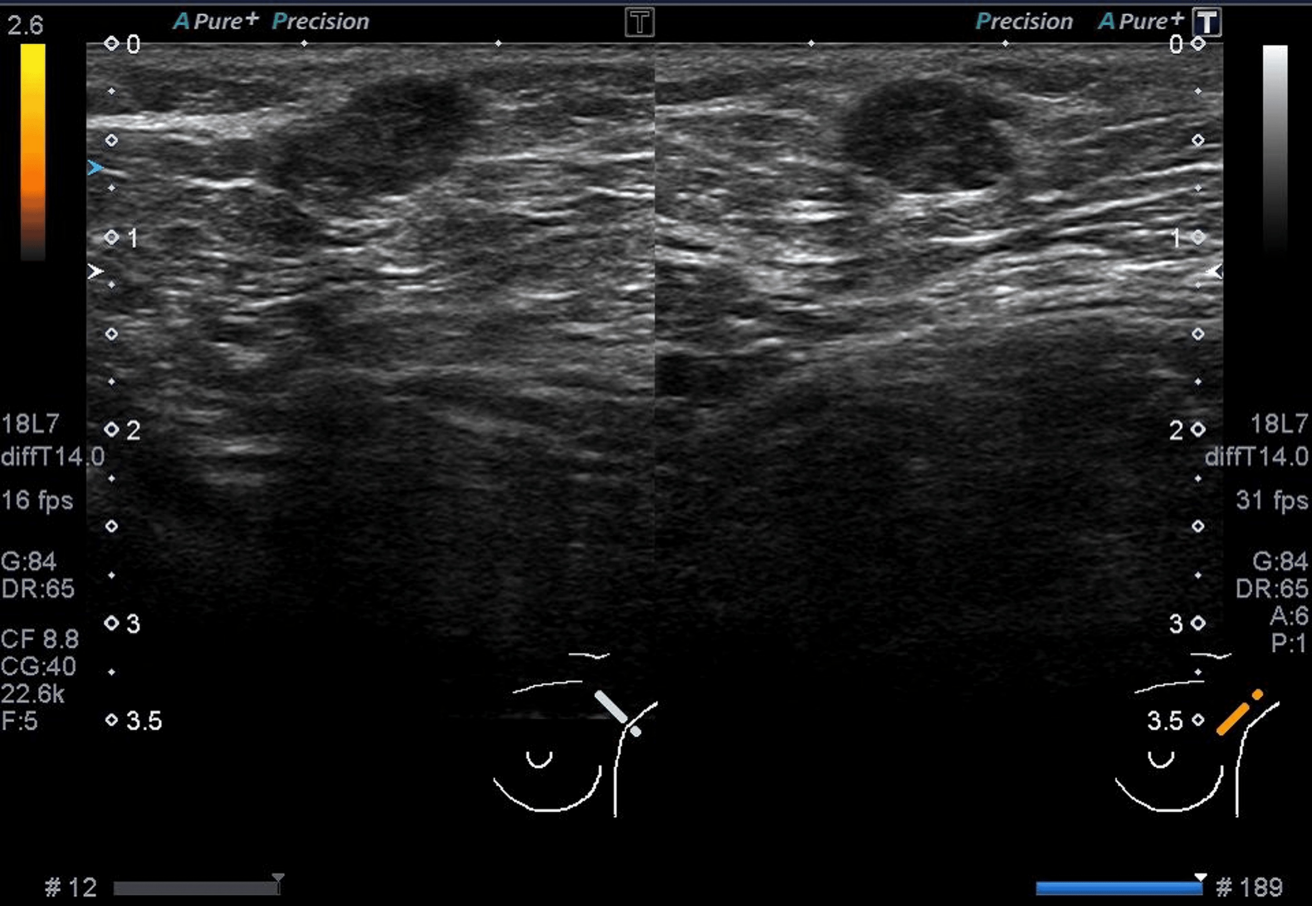
The mass after four months, now measuring 1.2 cm.

**Figure 3:**
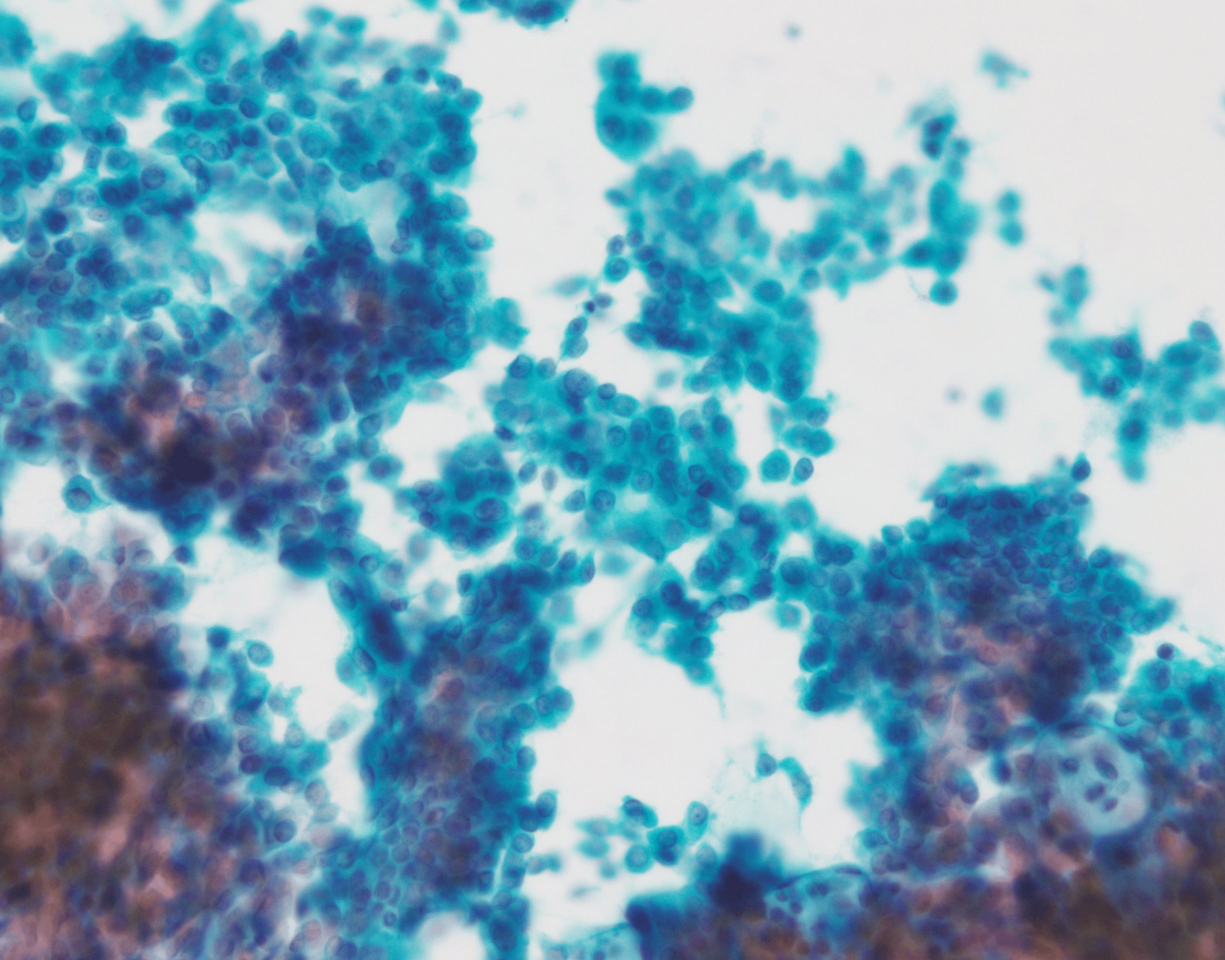
Cytological examination showing a Papanicolaou-stained smear revealing adenocarcinoma cells (magnification ×400).

However, after four months, the same mass had increased in size to 1.2 cm × 0.9 cm × 0.6 cm (Figure 2). The shape became slightly irregular with partially indistinct borders. This prompted the performance of a fine-needle aspiration cytology testing. Microscopically, the cellular clusters exhibit papillary, sheet-like, and wedge-shaped structures, with frayed edges observed at the margins. The nuclei show slight tension and a tendency for increased chromatin with fine granularity, which were suspicious for adenocarcinoma (Figure 3).

In light of the potential for breast cancer recurrence, a thorough systemic assessment was performed using computed tomography (CT) and positron emission tomography/CT (PET/CT) scans. Both examinations revealed an enhanced mass in the left axilla, while no other suspicious metastatic or recurrent lesions were identified. Considering the patient's history of bilateral breast cancer, genetic testing was conducted at this juncture, no mutations in the BRCA1/2 genes were detected.

**Figure 4:**
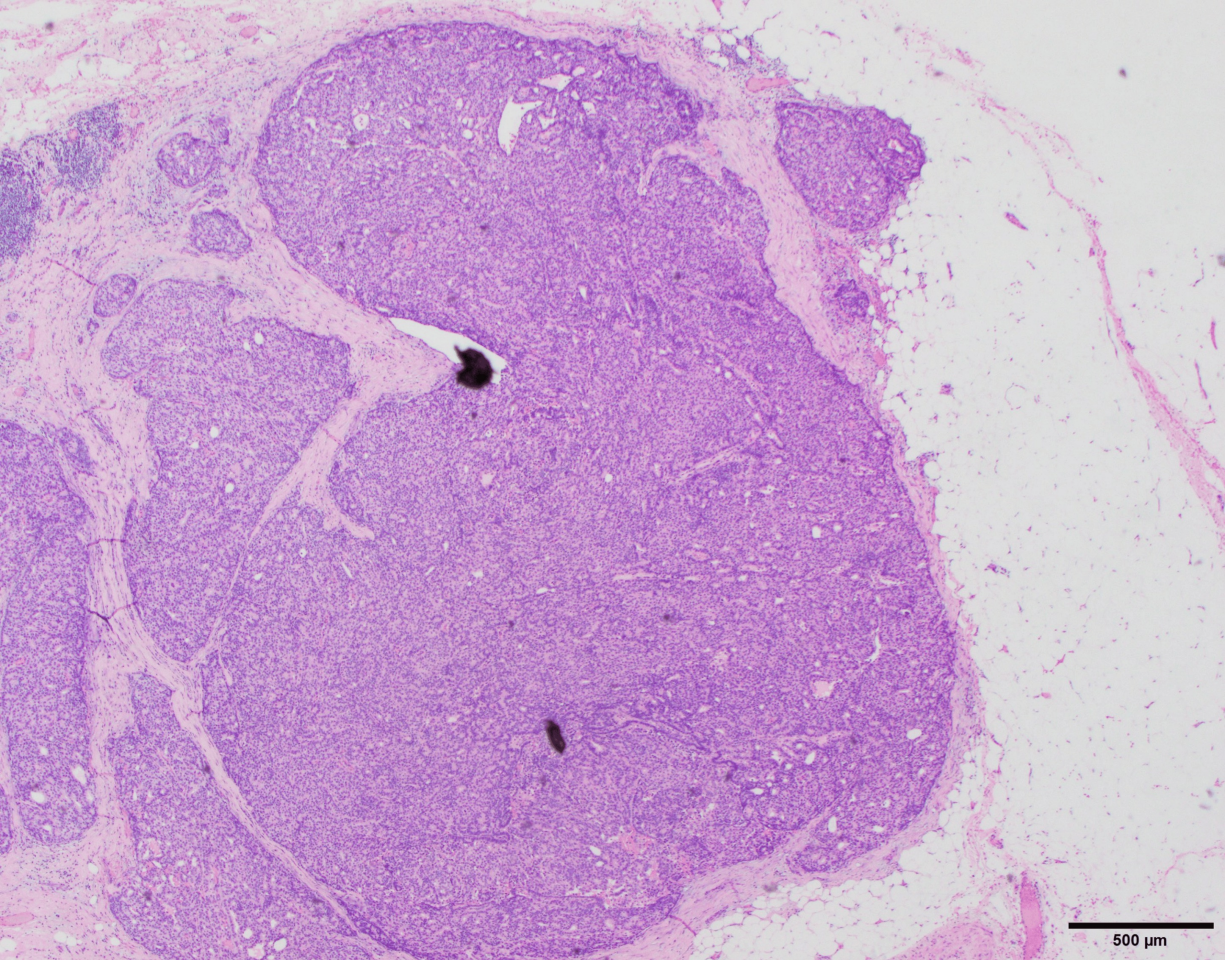
Pathological examination showing a relatively well-defined, oval, and lobulated ductal carcinoma measuring 0.6 × 0.5 cm (magnification ×20).

**Figure 5:**
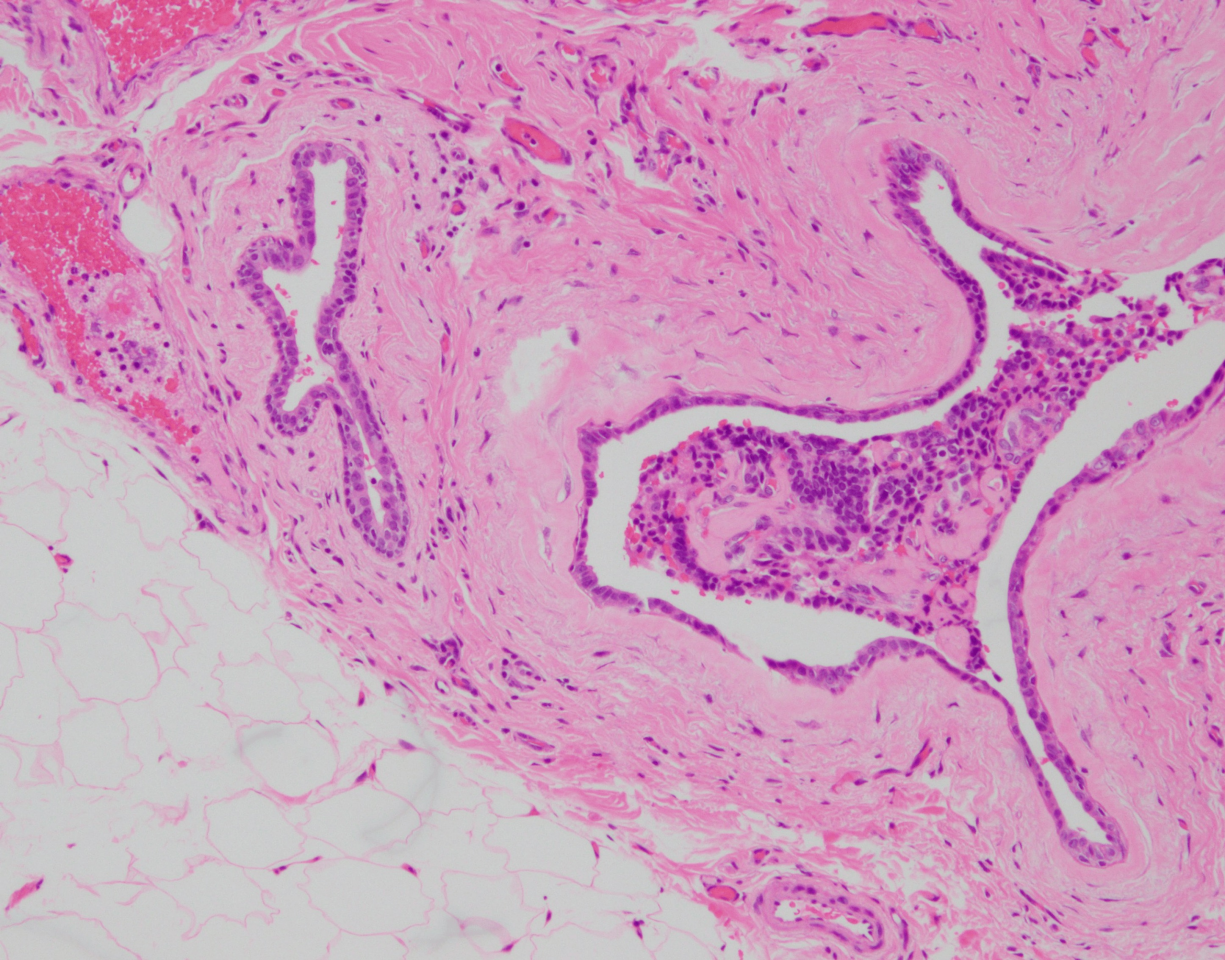
Non-neoplastic ducts surrounding the tumor (magnification ×40).

**Figure 6:**
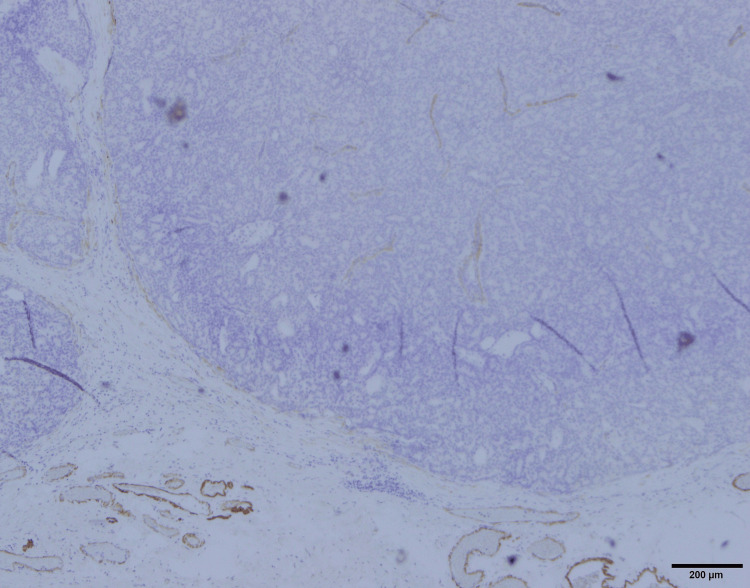
Most of the tumor cells lack myoepithelial cells (magnification ×20, Calponin staining).

In addition, owing to the possibility of a tumor originating from ectopic breast tissue in the axilla, a resection biopsy was performed, which included surrounding tissues several millimeters from the tumor. The tumor was an oval-shaped or slightly lobulated, relatively well-defined mass measuring 0.6 cm × 0.5 cm and was histologically characterized by cuboidal cells proliferating in solid nests (Figure 4). Non-neoplastic ductal structures were observed around the tumor, suggesting that the tumor originated from ectopic breast tissue (Figure 5). While the tumor proliferated within the ducts, the majority of the tumor-forming area lacked myoepithelial cells (Figure 6). Additionally, infiltration of irregular small nests into surrounding tissues was observed, leading to a diagnosis of encapsulated papillary carcinoma with invasion. It was Nottingham Grade Ⅰ, ER-positive, PR-positive, and HER2-negative, with a Ki67 index of 14%.

While the surgical margins did not reveal tumor exposure, a distance of approximately 200 μm from the tumor to the nearest margin was noted. Consequently, wide local excision (4.0 cm × 6.0 cm) was performed, encompassing the biopsy site. Due to the previous axillary lymph node dissection, sentinel lymph node biopsy was not conducted. The second surgical specimen also exhibited scattered breast tissue and intraductal carcinoma, but the margins remained negative. Accordingly, the patient was scheduled for ongoing monitoring while receiving hormone therapy, with particular attention on the assessment of local recurrence.

## Discussion

Ectopic breast tissue results from the failure of the mammary ridge to regress during embryogenesis [1,7,9-13]. EBC arising from this tissue is a rare condition, accounting for 0.3%-0.6% of all breast cancers [1]. The occurrence of both EBC and PBC, either synchronously or metachronously, as observed in this case, is extremely rare, with only five cases reported in the literature [2-5]. Additionally, there are no reports of cases occurring after bilateral breast cancer surgery based on our search. Although this tumor type is rare, we identified two important considerations regarding the diagnosis of axillary EBC.

The first point is that axillary ectopic breast tissue, which is situated just beneath the skin [8,13], is not usually included in the resection field during mastectomy and axillary lymph node dissection. Therefore EBC can develop after breast cancer surgery, as shown in this case. This superficial area of the axilla is not generally screened in routine breast cancer examinations or postoperative surveys. Given the superficial location of axillary ectopic breast tissue and its exclusion from standard mastectomy and lymph node dissection fields, incorporating routine ultrasound screening of the axillary skin in postoperative follow-ups could improve early detection of EBC [6,7]. Considering the low frequency of EBC, further research may be necessary to identify patients at higher risk for developing EBC in this context. In this report, only three cases of BRCA mutations have been investigated to date [5,8]. Further accumulation of data, including other risk factors for breast cancer, is required.

The second point concerns the US findings of EBC. Previous reports have often described these tumors as irregular, hypoechoic masses with unclear boundaries or speculations [10,11,13]. However, there are also cases, such as the present case, where the mass is oval-shaped with relatively clear boundaries. Additionally, there have been reports of high internal echogenicity in medullary and mucinous carcinomas arising from axillary ectopic breast tissue [6,14,15]. Therefore, it is reasonable to consider that, similar to PBC, US findings may vary depending on the histological architecture or type of breast cancer. There have also been reports of fibroadenoma and phyllodes tumors arising from ectopic breast tissue, with US findings similar to those of tumors occurring in the breast [16-18].

In axillary EBC, the presence of breast tissue surrounding the tumor in US is one of the characteristic findings [8]; however, this was not observed in the present case. The minimal amount of ectopic breast tissue surrounding the tumor, as observed histologically, is considered the primary reason for breast structures not being visible in US, and in this regard, the US findings also appear to reflect the tissue characteristics. Additionally, considering the patient's history of breast cancer and the uniform internal echogenicity of the tumor, a metastatic lymph node completely replaced by cancer cells was also considered in the differential diagnosis [8,19]. However, given that there were no lymph node metastases in the previous surgery and that the mass was situated directly below the axillary skin, this possibility was considered unlikely.

Previously, mastectomy was performed for EBC, but there is no evidence to support that mastectomy is superior to wide resection with clear margins, which includes ectopic breast tissue; therefore, according to the literature, wide resection is recommended [2,20]. Postoperative therapy was conducted by PBC protocols. Given the tumor's features (diameter of 0.6 cm, grade 1, ER-positive, PR-positive, HER2-negative, and Ki67 index of 14%, indicating a low risk of recurrence), only hormonal therapy is being administered.

## Conclusions

Following bilateral breast cancer surgery, a tumor developed just beneath the skin in the axilla. Postoperative screening of this site may aid in the early detection of EBC. Given the rarity of EBC, it is also important to accumulate data on high-risk factors for breast cancer, such as BRCA mutations.

US examination revealed an oval, hypoechoic, relatively homogeneous, and well-defined tumor. Pathologically, the tumor was an encapsulated papillary carcinoma with invasion, arising from ectopic breast tissue in the axilla. Its expansile growth correlated with the ultrasound findings. As with PBC, US can be highly useful in estimating the histological type and tissue structure of EBC.

## References

[1] Famá F, Cicciú M, Sindoni A (2016). Prevalence of ectopic breast tissue and tumor: a 20-year single center experience. Clin Breast Cancer.

[2] Sghaier S, GHalleb M, Marghli I, Bouida A, Ben Hassouna J, Chargui R, Rahal K (2021). Primary ectopic axillary breast cancer: a case series. J Med Case Rep.

[3] Hao JY, Yang CC, Liu FF (2012). Accessory breast cancer occurring concurrently with bilateral primary invasive breast carcinomas: a report of two cases and literature review. Cancer Biol Med.

[4] Oh SW, Lim HS, Lee JS, Moon SM, Park MH (2017). Invasive micropapillary carcinoma in axillar ectopic breast and synchronous ductal carcinoma in situ in the contralateral breast. J Breast Cancer.

[5] Addae JK, Genuit T, Colletta J, Schilling K (2021). Case of second primary breast cancer in ectopic breast tissue and review of the literature. BMJ Case Rep.

[6] Achouri L, Jellali A, Henchiri H, Boukhris S, Zaaimi Y, Mansouri H, Mahjoub N (2022). Primary ectopic breast carcinoma: a case report. J Med Case Rep.

[7] Zhang S, Yu YH, Qu W, Zhang Y, Li J (2015). Diagnosis and treatment of accessory breast cancer in 11 patients. Oncol Lett.

[8] Kim H, K EY, Han B-K (2022). Multicentric breast cancer of the axillary and pectoral breasts: a case report and literature review. J Breast Cancer.

[9] Sapon-Cousineau S, Moldoveanu D, Charpentier D, Gagnon A, Patocskai Patocskai (2022). Locally advanced breast cancer arising in the axilla. J Surg Case Rep.

[10] Harris MK, Guo MZ, Mangino A, Taylor C, Carson WE (2022). Sentinel node mapping and biopsy in ectopic axillary breast cancer: a case report and review of the literature. Clin Case Rep.

[11] Salemis NS (2021). Primary ectopic breast carcinoma in the axilla: a rare presentation and review of the literature. Breast Dis.

[12] Tsuji W (2020). Metachronous bilateral ectopic breast carcinoma in the axilla: a case report and literature review. Breast Dis.

[13] Lim HS, Kim SJ, Baek JM, Kim JW, Shin SS, Seon HJ, Heo SH (2017). Sonographic findings of accessory breast tissue in axilla and related diseases. J Ultrasound Med.

[14] Nihon-Yanagi Y, Ueda T, Kameda N, Okazumi S (2011). A case of ectopic breast cancer with a literature review. Surg Oncol.

[15] Nardello SM, Kulkarni N, Aggon A, Boraas M, Sigurdson ER, Bleicher RJ (2015). Invasive mucinous carcinoma arising in ectopic axillary breast tissue: a case report and literature review. Am J Case Rep.

[16] Tee SW, Tan YH, Jeyabalan D, Selvam D (2022). Fibroadenoma in axillary ectopic breast. BMJ Case Rep.

[17] Gajaria PK, Maheshwari UM (2017). Fibroadenoma in axillary ectopic breast tissue mimicking lymphadenopathy. J Clin Diagn Res.

[18] Oshida K, Miyauchi M, Yamamoto N, Takeuchi T, Suzuki M, Nagashima T, Miyazaki M (2003). Phyllodes tumor arising in ectopic breast tissue of the axilla. Breast Cancer.

[19] Jamaris S, Jamaluddin J, Islam T (2021). Is pre-operative axillary ultrasound alone sufficient to determine need for axillary dissection in early breast cancer patients?. Medicine (Baltimore).

[20] Verras GI, Mulita F, Tchabashvili L, Perdikaris P, Perdikaris I, Argentou MI (2022). Ectopic breast carcinoma. Prz Menopauzalny.

